# Comparison between 2,000 m and 3,000 m time trials to estimate the maximal aerobic speed for collegiate runners

**DOI:** 10.3389/fphys.2022.1005259

**Published:** 2022-09-30

**Authors:** Zonghao Du, Wei Lu, Diandong Lang

**Affiliations:** School of Strength and Conditioning Training, Beijing Sport University, Beijing, China

**Keywords:** maximal aerobic speed, 2,000 m time trial, 3,000 m time trial, field test, collegiate runners

## Abstract

Considered to be a lesser resource burden, 2,000 and 3,000 m time trials (TTs) have been recognized as alternatives to accurately estimate the maximal aerobic speed (MAS) derived from laboratory-graded exercise testing (GXT). Previous studies have commonly used ordinary least squares linear regression and the Bland–Altman method to compare the agreement between MAS and TT performance. The agreement analysis aimed to identify the systematic bias between the results of the two methods, rather than to identify similarities. The model II regression technique (ordinary least product regression) is increasingly favored by researchers in the field of physiology. Thus, we aimed to 1) use the ordinary least product (OLP) and bootstrap methods to determine the agreement between the average speed of 2,000 m TT (S2000) and the average speed of 3,000 m TT (S3000) and 2) determine whether S2000 or S3000 can accurately approximate the GXT-derived MAS. It is used as an alternative to estimate the MAS and prescribe training intensity. Thirty-five Beijing Sport University recreational male runners completed an MAS test in laboratory settings, followed by 2,000 and 3,000 m TTs randomly, with a 7-day interval. OLP regression was used to analyze the agreement between the GXT-derived MAS and S2000 and S3000. The bootstrap method was used to calibrate the equations. Differences between the GXT-derived MAS and S2000 and S3000 were compared using a one-way repeated measure analysis of variance (ANOVA) and a *post hoc* analysis (Bonferroni). The significance level was *p* < 0.05. The results showed that before calibration, the 95% CI of the OLP regression intercept and slope between the GXT-derived MAS and S2000 and S3000 did not include 0 and 1.00, respectively. These values, after calibration, included 0 and 1.00, respectively. *Post hoc* analysis revealed that S3000 closely approximated the GXT-derived MAS and underestimated 0.46% (0.06 km h^−1^ and *p* > 0.05), and S2000 overestimated 5.49% (0.81 km h^−1^ and *p* < 0.05) by the MAS. It concluded that the 3,000 m TT performance approximated the GXT-derived MAS compared to the 2,000 m TT performance. There exist fixed bias and proportional bias between the GXT-derived MAS and TT performance. More attention should be applied to calibration when using the TT performance to estimate the MAS.

## Introduction

A common and viable indicator to evaluate aerobic capacity and prescribe training intensity is VO_2_ max (Poole et al., 2008). However, there exist certain shortcomings in its practical application. VO_2_ max is not representative of the final athletic performance; runners with the same VO_2_ max have different race performances (Steve 2009). Moreover, expensive equipment and specialized technicians are required for laboratory-graded exercise testing (GXT) and expired gas analysis for obtaining VO_2_ max, adding difficulty to the assessment for most coaches and runners (Rafael et al., 2019; Lundquist et al., 2020). The running speed can be adjusted rather than the application of VO_2_ max (Abdullah 2021). Consequently, speed is prioritized by coaches to prescribe training intensity during practice (Steve 2009; Thomas et al., 2021; Manuel et al., 2022). Therefore, researchers and coaches suggest that more attention should be paid to performance rather than physical indicators (Steve 2009). The best descriptor of performance is the performance itself (Podlogar et al., 2022).

The maximal aerobic speed (MAS) represents the speed of movement produced by athletes at 100% VO_2_ max (Laurent et al., 2002), which integrates VO_2_ max and running economy. This variable can best explain the changes in the middle running performance over time (a runner with a higher MAS could have diminished decrements in performance or even improved performance) and represents a physiology descriptor that is 1) relatively practical to measure and 2) is a better predictor of performance than the currently widespread approaches (Jose et al., 2010; Podlogar et al., 2022). Several studies have demonstrated that the MAS can facilitate researchers to determine runners’ anaerobic speed reserve (Véronique Billat and Koralsztein 1996; Gareth, Paul, and Martin 2021), elucidate inter-individual differences (Véronique Billat and Koralsztein 1996), better reflect the training status, track the physiological development (Podlogar et al., 2022), and prescribe training intensity to improve aerobic performance (Daniel and Nathan 2015). Coaches and researchers have designed numerous tests for obtaining the MAS because of its importance in training.

With 100% MAS as the baseline, there exist MAS zones applied for aerobic training in running. Different training adaptations produced by MAS zones should be considered by recreational runners and coaches to achieve their goals. Continuous training sessions at a lower percentage of the MAS (70%–75% MAS, zone 2) can improve the running economy, and interval training sessions at a higher percentage of the MAS improve the running gait and MAS (95%–110% MAS, zone 5, zone 6) (Fernando et al., 2016). In addition, high-intensity aerobic methods used for MAS were developed by several researchers and have been validated, such as the maximal aerobic grid method (also termed the 100% MAS:70% MAS method), the Supramaximal Eurofit method, and the Tabata method. The differences between these methods majorly include the form of organization, rest ratio, training intensity of the MAS, and the duration applied. To achieve organized and efficient training, the priority while implementing these aerobic methods used by the MAS is to conduct an MAS test to determine the capability of each runner (Barker 2011).

The laboratory GXT using a gas analyzer system is considered the most accurate method to identify the MAS directly, where an exhaustive exercise protocol of 10–14 min should be completed by the runners under a monitored treadmill (Jesus et al., 2019). However, the original environment of athletic training cannot be replicated by laboratory GXT. Moreover, it is restricted by the demand for complex and expensive equipment because most coaches and athletes cannot afford it (Souza et al., 2014; Vincenzo et al., 2020; Abdulkerim et al., 2021). Thus, these disadvantages led coaches and researchers to focus on designing and validating field test protocols that can indirectly obtain the MAS with a lesser resource burden. Therefore, certain field tests are used in training and research. To derive the MAS, the University of Montreal Track Test was used to perform continuous incremental intensity testing protocols until fatigue was experienced by the runners (Luc and Robert 1980; Jesus et al., 2019). The multistage 20 m shuttle run test is one of the most commonly used tests in football and rugby because it contains intermittent movements of acceleration, deceleration, braking, and change of direction similar to those of ball events (Huerta Ojeda et al., 2020; Abdullah 2021). The results of these common field tests largely represent the completed stage or level rather than smooth and continuous pace speed values (Bellenger et al., 2015). Consequently, TT-based prediction for the MAS is more favorable by running events.

Estimates of the MAS based on the TT performance appear reasonable because TTs meet the specification of races in the athletic field and are efficient to conduct. The average speed of an ideal distance TT would be in agreement with or closely approximate the GXT-derived measure of the MAS (Souza et al., 2014; Bellenger et al., 2015). Several researchers have tried to estimate the MAS based on multiple TT distances such as 1,200 m (Rick et al., 2016), 1,500 m (Gareth et al., 2018), 2,000 m (Bellenger et al., 2015), and 3,000 m (Paul et al., 1997). Among these distances, 2,000 and 3,000 m TTs were especially related to the MAS and most favored by previous studies (Daniel and Nathan 2015; Bellenger et al., 2015; Paul et al., 1997, 5; Jose et al., 2010; William et al., 2018; Huerta Ojeda et al., 2020). However, the methods used by previous studies to conduct the agreement analysis between MAS and TT performance, including the paired *t*-test, correlation analysis, ordinary least squares linear regression, and Bland–Altman method, could not completely reflect the agreement between the two groups of data (Christian et al., 2009; Bellenger et al., 2015; Huerta Ojeda et al., 2020). The aim of an agreement analysis is to identify systematic bias between the results of the two methods rather than to identify similarities (Altman and Bland, 1983; Ludbrook, 1997). The model II regression technique (ordinary least product regression) is increasingly favored by researchers in the field of physiology (Ludbrook, 2010). Hence, this study compared the agreement between the GXT-derived MAS and 2,000 and 3,000 m TT performances and determine the difference between the GXT-derived MAS and TT performance for recreational collegiate male runners. The TT distance elicits an average speed closely approximating and estimating a GXT-derived measure of MAS such that the TT distance assesses the MAS regularly to identify modifications in the aerobic capacity and provides a valuable basis for training intensity prescription (Bellenger et al., 2015).

We used the ordinary least product regression (OLP) to compare the average speed of 2,000 and 3,000 m TTs in agreement with the GXT-derived MAS and establish the calibration equations. We assume that the 3,000 m TT may be completed at a speed closer to the GXT-derived MAS and validate it with one-way repeated ANOVA. We believe that the findings of the study will help coaches implement valid field tests for estimating the MAS.

## Materials and methods

### Participants

Thirty-five recreational collegiate male runners free from injuries and experienced in middle running voluntarily agreed to participate in this study (3,000 m performance: 12.3 ± 0.7 min, training history: 4.08 ± 1.17 years, and training frequency: four times/week) (personal data, Table 1). All participants signed the informed consent after understanding the research purpose, procedure, and underlying risks. No intensive exercise and drugs, caffeine, medications, or nutritional supplements that were known to influence the performance were consumed 48 h before attendance. The study was approved by the Ethics Commission of Beijing Sport University (No. 2022151H).

**TABLE 1:** Sample characteristics (mean ± SD) (*n* = 35).

Age (years)	Mass (kg)	Height (cm)	VO_2_ max (ml/kg^−1^/min^−1^)	Anaerobic threshold (%VO_2_ max, HR, and %HRmax)	Speed at anaerobic threshold (km/h)	MAS (km/h)
21.38 ± 1.34	73.41 ± 6.60	179.35 ± 4.84	51.27 ± 2.91	70% ± 8%	10.31 ± 2.53	15.11 ± 0.58
				150.49 ± 16.34		
				77.04 ± 7.91%		

### Procedures

General procedures: testing sessions comprised three consecutive periods (laboratory-individualized-graded exercise testing protocols and 2,000, and 3,000 m TTs) conducted in a random order, with a 7-day interval between the testing sessions. All testing sessions were performed under similar weather conditions (temperature 20–23°C and humidity 50%–60%). The participants performed the GXT in the laboratory, whereas 2,000 and 3,000 m TTs were conducted on an official 400 m track under similar environmental conditions (temperature 20–22°C, humidity 50%–60%, no rain, and light winds). A standardized 15 min warm-up was performed before the tests, including multiple dynamic stretch movements, including a knee-to-chest walk, walking quad stretch, hamstring hand walk-inchworm, and 6 min jog with an initial speed of 6 km/h^−1^, increased by 2 km/h^−1^ every 2 min to 10 km/h^−1^.

Laboratory-individualized-graded exercise testing protocol: the MAS and VO_2_ max were determined using this protocol. The automatic gas analyzer system software was used to measure the VO_2_ max and provide analysis results for every second time frame (CORTEX model MetaMax 3B, Leipzig, Germany). The highest average of VO_2_ for the last 30 s in any of the last steps was accepted as VO_2_ max. The major criterion for VO_2_ max determination was the existence of a “plateau,” an increment equal to or lower than 2.0 ml kg^−1^ min^−1^ in the last step or steps (Christian et al., 2009; Jose et al., 2010). If the participant does not reach the VO_2_ plateau, a retest was required. The evaluators completed the software calibration under the guidance of the manufacturer before the test. The respiratory data were processed and recorded using the software. All participants initiated a 10 min standardized warm-up and subsequently executed the GXT protocol with an initial speed of 12 km/h, which increased by 1 km/h every 2 min to 14 km/h and then by 0.5 km/h every 2 min until exhaustion. The inclination was kept constant at 1% to simulate the air resistance and assist participants in keeping an upright posture (Álvaro et al., 2020; Cerezuela Espejo et al., 2018). Participants were included in the analysis when the following criteria were met: 1) HRmax (maximum heart rate) > 180 beats/min, 2) the existence of the VO_2_ plateau, and 3) respiratory exchange ratio (RER) > 1.1 (Christian et al., 2009).

The ventilatory anaerobic threshold was automatically confirmed by the software of the metabolic cart based on changes in the ventilation volume, CO_2_ exhalation volume, and respiratory exchange rate. The VO_2_ was recorded automatically using the software of the metabolic cart, and the highest value was determined to be VO_2_ max if the previous inclusion criteria were achieved. The MAS was measured using the formula adapted by Kuipers: MAS (km.h^−1^) = S_f_+ (t/120*i) (Paul et al., 1997).

Here, S_f_ indicates the speed of the completed stage (km.h^−1^) after eliciting VO_2_ max, t represents the time spent running(s) of the uncompleted stage, and i denotes the increment of every stage (1 km h^−1^ increment to 14 and 0.5 km h^−1^ increment until exhaustion in the current study).

The heart rate data were monitored using Polar H10 and Polar M430 (Beijing, China) and synchronized using the gas analyzer system software. The rating of perceived exertion (RPE) was recorded immediately when participants were unable to maintain the required pace.

2,000 and 3,000 m TTs: all participants did their best to complete a 2,000 and 3,000 m TT alone on a 400 m outdoor running track and recorded the RPE value immediately after testing. Time was recorded with a manual stopwatch. Heart rate data were monitored using Polar H10 and Polar M430 (Beijing, China). The average speed of TTs was recorded as S2000 and S3000, which were compared by the GXT-derived MAS. There was no pace strategy requirement for participants.

### Statistical analysis

Data were analyzed using the R software (version 4.2.1). Ordinary least product regression (OLP) was used to compare the agreement between the MAS and S2000 and S3000, respectively. If the 95% CI for the OLP slope does not include 1.0, there was a fixed bias. If the 95% CI for the OLP intercept does not include 0, there was proportional bias (Ludbrook, 2010). OLP regression equations (MAS as a dependent variable and S2000 and S3000 as independent variables) were used to calibrate S2000 and S3000. Interval validation of the equation was performed using the bootstrap method. The agreement between the calibrated S2000 and S3000 and MAS was compared using the OLP.

The normal distribution for gathered data was examined through the Shapiro–Wilk test. Differences between the MAS and S2000 and S3000 were compared using a one-way repeated-measure ANOVA, and the significance level was *p* < 0.05. If *p* < 0.05, a *post hoc* analysis (Bonferroni) was used to measure the difference between the GXT-derived MAS and S2000 and S3000, respectively.

## Results

**FIGURE 1:**
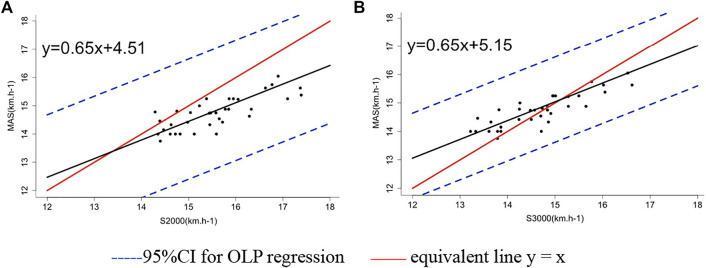
Ordinary least product (OLP) regression of the MAS on S2000 and S3000: the S2000 average speed of 2,000 m TT and the S3000 average speed of 3,000 m TT. **(A)** OLP regression of the MAS on S2000 **(B)** OLP regression of the MAS on S3000.

The slope of the OLP regression for MAS on S2000 was 0.65 (95% CI: 0.51–0.77), on S3000 was 0.65 (95% CI: 0.54–0.75), and the corresponding intercept was 4.59 (95% CI: 2.62–6.68) and 5.18 (95% CI: 3.57–6.84), respectively (Table 2). Although all measurements were within the 95% prediction interval of the OLP regression lines, both OLP regression lines intersected the equivalent line y = x (Figure 1). It shows that S2000 and S3000 underestimate the GXT value for low MAS but overestimate it for large MAS.

**TABLE 2:** Bootstrap internal validation of calibration equation results (*n* = 35).

	Calibration equation		Bootstrap (1,000 times)			
Index	Intercept α	Slope β	Mean of intercept α	95% CI	Mean of slope β	95% CI
S2000	4.59	0.65	4.53	2.62–6.68	0.66	0.51–0.77
S3000	5.18	0.65	5.15	3.57–6.84	0.65	0.54–0.75

Using the MAS as the dependent variable and S2000 and S3000 as independent variables, the calibration equation for the OLP regression is MAS = 0.65 * S2000 + 4.59, MAS = 0.65 * S3000 + 5.18. The means and 95% CI of slopes and intercepts of the bootstrap sample calibration equations (1,000 times) are shown in Table 2.

The slope of the OLP regression of the calibrated MAS (95% CI) is 1.00 (0.91–1.10) and 1.00 (0.92–1.09), respectively and the intercept (95% CI) is 0 (−2.52 to 2.29) and 0 (−2.98 to 2.73), respectively.

The one-way repeated-measures ANOVA is determined to be statistically significant (*p* < 0.05).

The results of *post hoc* analysis (Bonferroni) show there was a significant difference between the MAS and S2000 (*p* < 0.05), and no significant difference was observed between the MAS and S3000 (*p* > 0.05), as shown in Table 3.

**TABLE 3:** Difference between the MAS and S2000 and S3000.

	GXT-MAS	2,000 m	3,000 m
Speed (km/h)	14.74 ± 0.58	15.55 ± 0.89	14.68 ± 0.89
Difference (95% CI)		−0.81* (−1.05 to −0.57)	0.06 (−0.15 to 0.27)
HRmax (beats/min)	194.77 ± 6.43	197.17 ± 4.69	197.03 ± 5.45
RPE	18 ± 0.77	17.77 ± 0.6	18.09 ± 0.61

*Significant difference from GXT-MAS (*p* < 0.001).

## Discussion

For reasonable load protocols, participants with good training experience familiarized with the experimental protocol ensured that they could tolerate high-load stimuli, reach their maximal potential, and elicit true MAS in the experiment. In this way, the test–retest reliability of the experiment is warranted (Alvero-Cruz et al., 2017; Saddek et al., 2020; Álvaro et al., 2021). In our experiment, all participants were familiarized with protocols before the formal experiment and contributed maximally (HRmax > 193 beats/min, RPE > 18, RER > 1.1, and all reached VO_2_ plateau). The VO_2_ plateau appears in the GXT protocol, meaning that all participants reached VO_2_ max. This is due to two fundamental reasons: first, the participants have a good training experience, allowing them to tolerate a high-load stimulation. Second, the GXT protocol is relatively reasonable, with each participant being able to reach exhaustion. The GXT lasting 620 s caters to the completion time, which falls within the duration ranging between 600 and 840 s recommended by the previous study. Shorter GXT protocols lasting 600–840 s permit the participants to reach their maximal potential not limited by accumulative fatigue of muscles and the cardiovascular system (Véronique Billat and Koralsztein 1996; Jesus et al., 2019). Additionally, the length of time for 2,000 and 3,000 m TTs in this study was 460 and 730 s, respectively, both of which ensured that participants performing at a high level of aerobic speed even reached the VO_2_ max (Jose et al., 2010; Lundquist et al., 2020).

The present study analyzed the agreement between the MAS and the average speed of 2,000 and 3,000 m for recreational collegiate male runners. The results of the study will provide reference data and methods for accurately estimating the MAS with an average speed of 2,000 versus 3,000 m. Commonly used methods used previously included the paired *t*-test, correlation analysis, ordinary least squares linear regression, and Bland–Altman method (Álvaro Cristian et al., 2020; Bellenger et al., 2015; Christian et al., 2009). The paired *t*-test can only assess whether the means of the two groups of data are equal, and correlation analysis can only assess the degree of the simultaneous changes between the two groups of data, which cannot completely reflect the agreement between the two groups of data (Altman and Bland, 1983). The aim of the agreement analysis is to identify the systematic bias between the results of the two methods rather than to identify similarities (Ludbrook, 1997). Systematic bias can be further divided into fixed bias and proportional bias. Fixed bias implies that one method provides higher (or lower) values across the whole range of measurement, and proportional bias implies that one method provides values that diverge progressively from those of the other (Ludbrook, 1997; Ludbrook, 2010).

The determination of fixed bias and proportional bias is often compared with the equivalence line y = x through the ordinary least squares regression line. However, the OLS is theoretically not suitable to compare the agreement between the results of the two methods because the OLS method requires that the x values are fixed by study design, whereas both y and x values are usually free to vary and are subject to error. In this case, model II regression techniques (OLP) must be used (Ludbrook, 2010). Bland et al. have demonstrated that the OLP is more suitable for comparing and calibrating the agreement between the two methods. Furthermore, the Bland–Altman method is the classical method of agreement analysis, which has the merit of directly quantifying the concentration level and dispersion level between the two methods. However, when there is a proportional bias, the mean value of the difference and the 95% agreement boundary derived by the Bland–Altman method are inaccurate (Ludbrook, 1997), and this method cannot calibrate the measurement results. The GXT method used in this study is considered the reference standard for MAS measurement. The existence of measurement errors cannot be completely excluded. Therefore, the OLP regression method recommended by Ludbrook et al. was used to analyze the agreement between the GXT-derived MAS and TT performance and calibrated the equation.

The OLP regression results revealed a proportional bias between the GXT-derived MAS and TT performance before calibration. The TT performance tended to underestimate the GXT-derived MAS for lower MAS and overestimates it for large MAS. The presence of proportional bias indicated that lower GXT-derived MAS is underestimated by TT performance and higher GXT-derived MAS is overestimated. In addition, there was a fixed bias between the GXT-derived MAS and TT performance before calibration, and the size of the fixed bias was affected by the proportional bias (Ludbrook, 1997). The abovementioned results indicate the poor agreement between the GXT-derived MAS and TT performance.

The bootstrap sampling method (1,000 times) was used to validate the calibration equations. Both the calibrated GXT-derived MAS and TT performance were free of fixed and proportional bias, indicating that the calibrated equations improved the agreement between the GXT and TT, suggesting the need to calibrate the equations for using TT performance to estimate the GXT-derived MAS.

*Post hoc* tests for one-way repeated-measures ANOVAs showed the 3,000 m TT performance closely approximated the GXT-derived MAS and underestimated 0.46% (0.06 km h^−1^, *p* > 0.05), and the 2,000 m TT performance overestimated 5.49% (0.81 km h^−1^, *p* < 0.05) by the GXT-derived MAS. Consequently, the 2,000 m TT enables the runners to elicit an average pace that exceeds the MAS (the average speed of 2,000 m TT was 105% of the GXT-derived MAS), requiring more anaerobic contribution than that of the 3,000 m TT distance (Lundquist et al., 2020). Numerous studies have identified that S3000 ranged from 97% to 101% MAS (mean 100%), and the ratio between S3000 and the MAS was approximately 1 (Véronique Billat and Koralsztein 1996; Jose et al., 2010; William et al., 2018; Rafael et al., 2019). In our study, S3000 was 99% of MAS, suggesting that the 3,000 m pace employed 100% of the VO_2_ max (Viswanath et al., 1995; Jose et al., 2010), requiring the highest contribution of aerobic speed understandably correlated with the MAS. Therefore, 3,000 m can be considered as the critical distance to generate the maximal aerobic speed. The TT distance became gradually less than 3,000 m, the contribution of anaerobic capacity started to be more important, and the ratio between the TT performance and MAS increased progressively.

Additionally, the day-to-day variations in environmental conditions and the fatigue of the last test possibly affected the TT performance and laboratory MAS measurement (Podlogar et al., 2022). Environmental conditions should be maintained during data collection (temperature 20–22°C and humidity 50%–60%) with randomization of the testing order with at least a 7-day interval between sessions. The alone running method reduces the competition interference between the participants with plenty of experience in TTs, ensuring a smooth pace strategy. Consequently, the effect of weather conditions, fatigue-induced impact, and competition interference were balanced.

The study had a few limitations: 1) Due to a limited sample, no further screening was performed for participants. Further studies could be conducted by expanding the sample to validate the calibration equations. 2) No specialized equipment was used to measure the wind speed, although TTs were conducted on an official 400 m outdoor track with no rain and light winds. 3) The variations in the MAS and physiological parameters depend on the subtle parameter of laboratory GXT protocols (incremental speeds and duration, 0.5–2 km h^−1^ increase in the speed for every 30 s to 6 min of work). A review suggested that these parameters led to a 10% variance in the MAS (Véronique Billat and Koralsztein 1996; Lundquist et al., 2020). Thus, the MAS is considered to be a contrived variable decided by the employed GXT protocol. Given the differences in the subtle parameter in protocols deriving the MAS, it remains unknown whether the research results can be extrapolated using MAS measures in different protocols. The variation in the MAS obtained in different laboratory GXT protocols within different levels of runners need additional research for confirmation. 4) Participants will have a large individual variation in lactate response at the same protocol, indicating that the two runners train at a fixed intensity, out of which one is below the anaerobic threshold; however, the other is above. This will significantly affect the energetics of the workout. Blood lactate concentration should be added to all tests to accurately observe individual metabolic strain in response to the same protocol (Steve 2009).

## Conclusion

This study indicated that the 3,000 m TT performance approximates the GXT-derived MAS compared to the 2,000 m TT performance. There are fixed bias and proportional bias between the GXT-derived MAS and TT performance, meaning poor agreement between the GXT-derived MAS and TT performance. More attention should be paid to calibration when using TT performance to estimate the GXT-derived MAS.

## Data Availability

The original contributions presented in the study are included in the article/Supplementary Material; further inquiries can be directed to the corresponding author.
